# Long-term follow-up of an attenuated presentation of NAXE-related disease, a potentially actionable neurometabolic disease: a case report

**DOI:** 10.3389/fneur.2024.1204848

**Published:** 2024-02-14

**Authors:** Montaha Almudhry, Chitra Prasad, C. Anthony Rupar, Keng Yeow Tay, Asuri N. Prasad

**Affiliations:** ^1^London Health Sciences Centre and Western University, London, ON, Canada; ^2^Department of Neuroscience, King Fahad Specialist Hospital, Dammam, Saudi Arabia; ^3^Department of Pediatrics, Section of Genetics and Metabolism, Western University, London, ON, Canada; ^4^Department of Pathology and Laboratory Medicine, Western University, London, ON, Canada; ^5^Department of Medical Imaging, Western University, London, ON, Canada; ^6^Departments of Pediatrics and Pediatric Neurology, Western University, London, ON, Canada

**Keywords:** PEBEL1, *NAXE gene*, NAD(P)HX epimerase, niacin, cerebellar ataxia

## Abstract

**Background:**

Early-onset progressive encephalopathy with brain edema and/or leukoencephalopathy (PEBEL-1) is an autosomal recessive disorder whereby a fluctuating clinical course is exacerbated by febrile illnesses. Pathogenic NAD(P)HX epimerase (*NAXE*) gene mutations underpin this disorder. This mutation damages the metabolite repair system involved in regenerating crucial redox carriers. Longer survival has rarely been reported in this potentially actionable entity.

**Objectives:**

This case study aims to report a milder phenotype of a patient with *NAXE* gene mutation and his longitudinal follow-up of more than 20 years.

**Case report:**

A 24-year-old man first became symptomatic in infancy with frequent initial neurological decompensations in the setting of infections with subsequent clinical improvement followed by stability with residual cerebellar dysfunction. Clinical features noted over the years include chronic ataxia, nystagmus, ptosis, mild spasticity of lower limbs, and neuropsychiatric symptoms. Cerebellar and spinal cord atrophy were noted in cranial and spinal MR imaging. Biallelic homozygous variants in the *NAXE* gene (c.733 A>C) were identified on whole exome sequencing. Symptom management included the initiation of a mitochondrial cocktail with carnitine, coenzyme Q, and thiamine. Subsequently, niacin (Vitamin B3), which is involved in the cellular biosynthesis of NAD+, was added, given its potentially beneficial therapeutic impact.

**Conclusion:**

A missense homozygous variant in the *NAXE* gene is described in this patient with a milder clinical phenotype of the disease. Supplementation with niacin in addition to a mitochondrial cocktail presents a potential supportive therapeutic option to reduce disease progression.

## 1 Introduction

A pathogenic biallelic mutation in the *NAXE* gene causes early-onset progressive encephalopathy with brain edema and/or leukoencephalopathy-1 (PEBEL1) (OMIM # 617186). The course of PEBEL1 tends to be lethal, with rapid deterioration and death following even trivial fever or infection in childhood (1–4). The condition is attributed to a failure of the metabolic repair pathway, requiring the participation of NAD(P)HX epimerase, an enzyme encoded by the *NAXE* gene. The entity calls into focus the critical role played by NAD and NADP in vital cell processes. We report here an attenuated milder phenotypic presentation of PEBEL-1 presenting in infancy and followed through to young adulthood, suggesting considerable variability in the natural history of this potentially actionable disorder.

## 2 Case presentation

**Figure 1:**
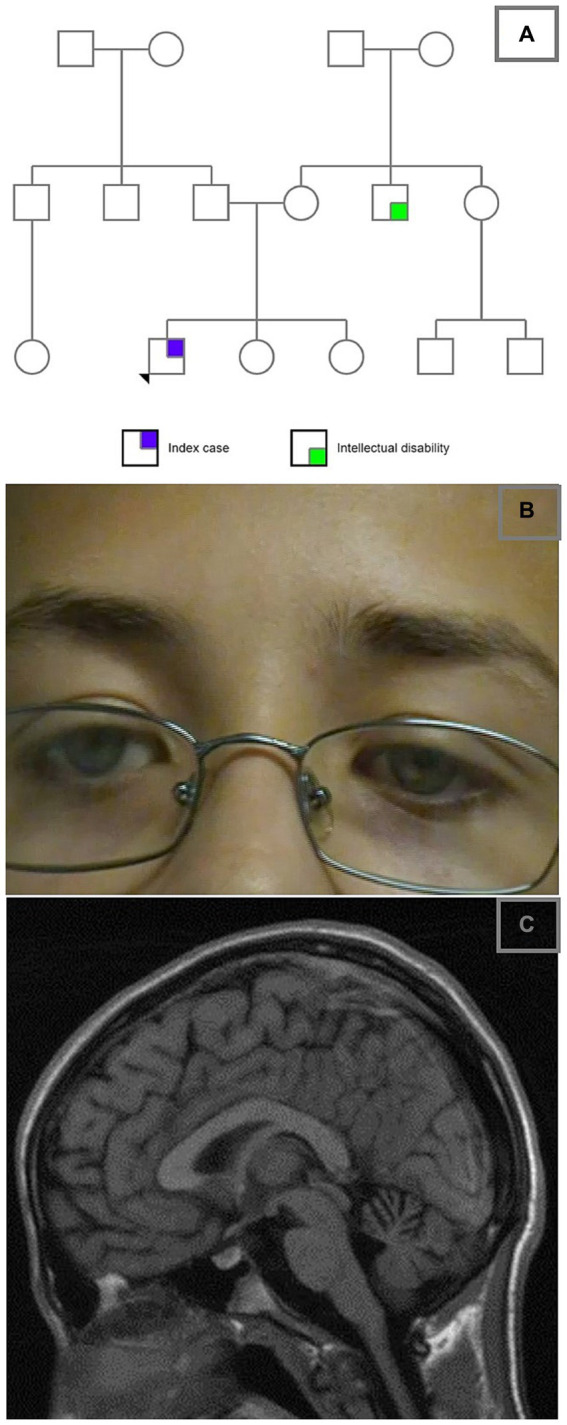
**(A)** Family pedigree; **(B)** bilateral ptosis; **(C)** sagittal T1 weighted MRI image demonstrating superior cerebellar vermis atrophy.

The proband, a 24-year-old man of South African descent, was the first child born to a healthy mother. There is no known parental consanguinity. Pregnancy was uneventful, and the infant was delivered by cesarean section due to failure to progress. The patient had two younger healthy siblings without any family history of neurologic symptomatology (Figure 1A). Birth weight was noted to be 2.9 kg, and no perinatal risk factors or postpartum complications were reported. His early developmental milestones were attained and deemed normal up until the age of 15 months, when an abrupt onset of neurological decline presented with ocular strabismus, head tilt, and nystagmoid eye jerks noted a few days following an ear infection. Thereafter, he went on to experience episodes of ataxia, confusion that would last between minutes and hours, and regression in his baseline motor and language skills. These episodes would be followed by a partial recovery that was incomplete and never to the neurological baseline state prior to the onset of illness. Early in the course, these episodes would vary in frequency, occurring once every 1–4 weeks. These events were generally, but not always, associated with infections and febrile illnesses. Assessment at the age of 2 years and 5 months disclosed developmental delay with a developmental age closer to that of a 1-year-old. By this age, the patient was crawling but had not achieved walking and had no spoken language. His language comprehension, however, remained advanced when compared to his expressive skills.

The clinical course was punctuated by episodes of neurological decompensation until 2.5 years of age. These decompensations were characterized by ophthalmoplegia (mostly vertical), ptosis, gait ataxia, lethargy, and developmental regression and incoordination (Figure 1B). He would have difficulty speaking and would be unable to participate in sporting activities of which he was previously capable. The clinical cessation of his episodes coincided with the empiric introduction of a mitochondrial cocktail of L-Carnitine 330 mg once daily, coenzyme q10 200 mg twice a day, B-100 complex once daily (100 mg of vitamins B1, B2, B3, B5 (pantothenic acid), and inositol, 10 mg of bioactive vitamin B6, 1,000 mcg of folic acid, 10 mg of choline, 500 mcg of bioactive vitamin B12, and 300 mcg of biotin in each tablet), and riboflavin 200 mg once daily, resulting in dramatic observed improvements in his neurodevelopmental status with residual ataxia and the stabilization of his neurological status. Aside from this, the patient received surgical correction for strabismus but continued to have gaze-evoked nystagmus. Hearing ability was subjectively normal, and he had never reported seizures, abnormal movements, or dystonic posturing. Kyphoscoliosis and longstanding reports of constipation were also noted, the latter possibly being related to gastrointestinal dysmotility.

Throughout childhood, our index case continued to display clumsiness with particular concerns around balance and limb coordination. He was noted to have a staggering and stumbling gait, difficulties using utensils independently, and challenges with his language skills. He also experienced intermittent fatigue with a tendency to do poorly during intercurrent infections; however, he continued to perform satisfactorily with his academic work at school. Neuropsychological assessment at the age of 8 years documented his cognitive performance within a normal range. Throughout childhood and adolescence, he was diagnosed with generalized anxiety, which was managed by citalopram. At 24 years of age, the patient was admitted to the mental health unit after experiencing violent intrusive thoughts consistent with obsessive-compulsive disorder as well as delusions in the setting of psychosocial stressors. Beyond the teenage years, he was neurologically stable, and at the time of the study, he was majoring in accounting and attempting to develop life skills for independent living.

On examination at the age of 24 years, the patient was aware and engaging. His speech was of a slurred quality but was otherwise fluent. His responses to questions appeared mildly sluggish but with no apparent intellectual difficulties; a formal assessment was not considered necessary in view of his current abilities and cognitive functioning. His examination identified notable difficulties in initiating saccades, often with an overshot, while ocular pursuit movements were incomplete or broken up by intrusions. In primary gaze, he had square wave jerks and bilateral ptosis. Limitations in his extraocular muscle movements were observed particularly during supraduction and infraduction. Muscle tone was low in the upper extremities, and spasticity was noted in the lower extremities with intact strength and positive pyramidal signs. Gait examination showed difficulty in tandem gait with a tendency for toe walking in stressed gait maneuvers. On examination of the spine, kyphoscoliosis was evident. Sensory examination showed distal impairment of vibration sense, but pain sense, temperature sense, and joint position sense were preserved. Past pointing in finger-nose testing was not apparent, but there were slowed and rapid alternating movements. Supplementary Video S1 demonstrates the patient’s physical findings.

A provisional diagnosis of mitochondrial disorder was made initially based on ophthalmoplegia, recurrent episodes of regression, and the fluctuation of symptoms with intercurrent illness. Later, hereditaory progressive ataxias were also amongst the differential given the longstanding course of ophthalmoplegia and the predominance of cerebellar involvement.

Extensive investigations were performed to uncover the possible underlying inborn error of metabolism, revealing an elevated CSF lactate at 3.0 mmol/L, while the rest of the metabolic testing came back unremarkable, including plasma lactate, ammonia, urine organic acids, amino acid profiles, CK, α-fetoprotein, vitamin E, uric acid, free and total acylcarnitine profile, homocysteine, 7-Dehydrocholesterol, cholesterol esterification analysis for Niemann-Picks C disease, glucocerebrosidase, galactosidase, beta-glucosidase, peroxisomal studies, skin biopsy, and fibroblast culture for the activity of pyruvate dehydrogenase. Imaging studies showed a minor delay in myelination on the initial MRI study (not accessible at the time of presentation to the tertiary care clinic), while a subsequent imaging study performed at the age of 13 years showed moderate atrophy of the superior vermis and diffuse prominence of cerebellar folia (Figure 1C). An MRI of the cervical spine demonstrated a relatively thin thoracic spinal cord. MRS detected a reduced NAA peak in the cerebellum. No evidence of peripheral neuropathy or myopathy was demonstrated on the NCS/EMG. A complete X-ray of the spine confirmed right lower thoracic and lumbar scoliosis of approximately 50 degrees centered at T11-12.

An extensive set of genetic investigations was conducted, including FMR1, NPC1/2, SPG 7 genes, multi-gene panels for spinocerebellar ataxia, and mitochondrial genome sequencing, all of which were inconclusive. Whole exome sequencing revealed a homozygous variant in the *NAXE*: c.733 A>C in exon 6 (p.Lys245Gln), which was inherited from both parents. This sequence change replaces lysine, which is basic and polar, with glutamine, which is neutral and polar, at codon 245 of the NAXE protein (p.Lys245Gln). This rare variant has an allele frequency of 2.48 E-5 in the gnomAD database. *In silico* analyses of the c.733 A>C variant using SIFT, Polyphen-2, Mutation Taster, and Panther predicted not tolerated, probably damaging, disease-causing prob.: 0.9999, and probably damaging, respectively. Further *in-silico* analysis was conducted by submitting the protein variant to ProtVar^1^ and Dynamut^2^ webtools for effects on Protein structure and stability. The genomic effects of c.733 A>C missense mutation disclosed a CADD phred-like score of 28.9 (probably deleterious as a CADD phred score above 20 is considered significant) while the effect on protein structure with the EVE (evolutionary model of variant effect) score is considered pathogenic with loss of catalytic site (*p* = 0.01) at p.Lys245Gln and loss of methylation at the same site (*p* = 0.02). The Dynamut analysis also predicted a destabilizing effect (ΔΔG: −0.403 kcal/mol). These data are summarized in Table 1. Thus, there is strong evidence that the genomic variant has significant negative effects on protein structure. The variant was classified as a variant of unknown significance (VUS) in the databases, mainly based on the lack of evidence to determine the role of this variant in disease. There has been a report of a homozygous patient with the same NAXE variant (5), and another compound heterozygous NAXE patient was identified in a series of patients with Leigh syndrome (6). We classify the NAXE c.733A>C variant as ACMG 2-probably pathogenic.

**Table 1:** Summary of *in-silico* analysis of the mutational effect supporting pathogenicity.

Gene	*NAXE*
Cytogenetic band	1q22
Exon	Exon 6
DNA change	c.733 A>C
Protein change	p.Lys245Gln
Protein subcellular location	Mitochondrion
Protein name	NAD(P)H-hydrate epimerase
Mutation	Missense
Zygosity	Homozygous
Inheritance	Autosomal recessive disease
OMIM number	617,186
Computational *in silico* evidence Algorithms used: SIFT, PolyPhen-2, Align-GVGD ProtVar Dynamut-prediction outcomes	l likely disruptive *CADD phred-like score*: 28.9 (probably deleterious) *EVE score*: 0.86 (pathogenic) *Preservation time*: 1628 (million years), Pdel:0.89 (probably damaging) (https://www.ebi.ac.uk/ProtVar/query?search=Q8NCW5%20K245Q) *NMA-based and other structures-based prediction*: destabilizing effect

CADD, Combined Annotation-Dependent Depletion; EVE, Evolutionary model of Variant Effect; Pdel, Probability of deleterious effect; NMA, Normal mode analysis.

**Figure 2:**
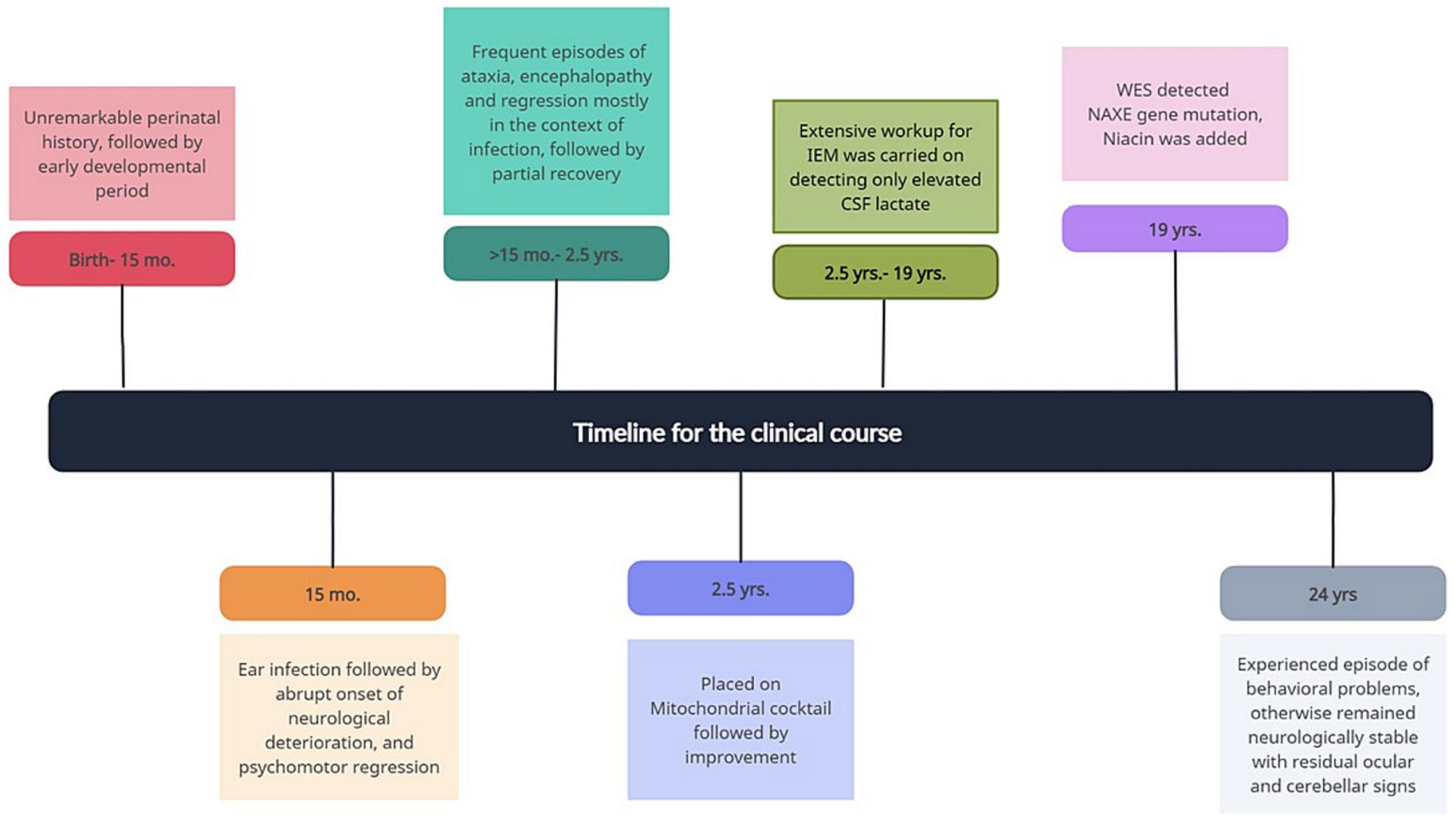
The timeline of the clinical course.

Following diagnosis, niacin 40 mg twice daily was added to his mitochondrial cocktail, with good tolerability and no adverse effect. Figure 2 presents the timeline of the clinical course.

## 3 Discussion

PEBEL1 (OMIM# 617186) is an autosomal recessive neurodegenerative neurometabolic disorder caused by a pathogenic mutation in the *NAXE* gene (formally known as the APOA1BP gene) on chromosome 1q22. This gene encodes for apolipoprotein A-I-binding protein that catalyzes the epimerization of the S and R forms of NAD(P) HX.

This process, along with other critical metabolite repair systems in the body, has become an emerging field of inborn error of metabolism (IEM). Dysfunction of the mitochondrial NAD(P)HX repair system translates clinically to this devastating neurogenetic disease (7, 8). Nicotinamide adenine dinucleotide (NAD) and nicotinamide adenine dinucleotide phosphate (NADP) are redox equivalents that play important roles in numerous physiological functions, such as catabolic and anabolic reactions, energy production, biosynthetic pathways, and antioxidant defense. The related hydrates R-NAD(P)HX and S-NAD(P)HX and cyclical NAD(P)HX are formed as a result of spontaneous or enzymatic hydration of the nicotinamide ring of NADH or NADPH. In low-pH and high-temperature conditions, spontaneous hydration is more evident, resulting in neurotoxic metabolites that cannot act as electron donors or acceptors and hinder various dehydrogenases (9, 10).

To eliminate the development of the damaging byproducts, the nicotinamide nucleotide repair method utilizes two conserved ADP- or ATP-dependent enzymatic mechanisms: NAXD (NAD(P)HX dehydratase), which recovers NAD(P)HX by working primarily on its S epimer, and NAD(P)HX epimerase (NAXE), a second enzyme that transforms NAD(P)HX between its S and R forms and serves as the substrate for NAXD. This pathway is crucial to prevent the accumulation of toxic metabolites and the inhibition of multiple cytosolic dehydrogenases that are important for the maintenance of cell metabolism and homeostasis (Supplementary Figure S1) (7, 11).

There are only a few case series in the literature describing this entity. The described patients typically present during the infantile period with rapidly progressive neurologic decline after a period of normal development. The disease typically begins with a febrile sickness or infection, which is followed by abrupt onset of ataxia, psychomotor regression, hypotonia, respiratory insufficiency, seizure, vegetative state, coma, and death in the first years of life. Survivors experience fluctuating trajectory of illness, incremental disability associated with tetraparesis, and loss of ambulatory capacity, eventually becoming bedridden and dependent on care providers for all activities of daily living (1). Skin lesions and cardiac involvement were also reported among the extra CNS manifestations. Brain MRI findings are variable across different reported subjects with described symmetric deep white matter abnormalities in periventricular distribution, symmetric hyperintensity in the bilateral middle cerebellar peduncles, brain edema, brain atrophy, and cerebellar changes (12). Increased CSF lactate has been reported in some patients; nevertheless, metabolic testing has been found to be of low diagnostic value in the majority of cases (1, 2, 4, 11, 12). In a study by Jin Sook Lee et al. examining genetic variability in 61 patients with Leigh syndrome at Seoul National University Children’s Hospital through mitochondrial genetic analysis followed by whole-exome sequencing, the *NAXE* gene was recognized among the newly identified nuclear genes associated with features of Leigh syndrome (6).

In the aforementioned patient, the disease was provoked by intercurrent febrile events similar to the previously reported cases; the disease course, however, followed a steadily improving path from the age of 2 years, followed by a plateauing phase and stabilization beyond the teenage years. Despite relative clinical stability, radiological progression continued in our patient, predominantly affecting the cerebellar vermis and hemispheres, in keeping with the patient’s residual ataxic gait. The same genetic variant in the *NAXE* gene: c.733 A>C (p.Lys245Gln) has been mentioned before in the literature with more severe phenotypes, unlike our patient, probably pointing to poor genotype-phenotypic correlation, and requires a more cautionary approach to prognostication (1, 5). As in the reported cases, our patient underwent a multitude of metabolic, genetic, and histopathological studies with unrevealing results. Subsequent diagnosis was achieved through whole exome sequencing highlighting the essential role of exome sequencing in uncovering rare disorders and calling for earlier arrangement of such studies. Despite the current classification of this variant as a VUS and the lack of an official ACMG1/2 classification, a published case series involving two patients with homozygous mutations and one patient with heterozygous mutation, who are described to have NAXE-related disease, and the multiple lines of evidence from *in-silico* analysis presented here strongly support the pathogenicity of this variant (13).

In the reported case series by Trinh et al., two late-onset PEBEL cases are described. The first was diagnosed at the age of 20 years and passed away 2 years later with decompensation triggered by alcohol and tetrahydrocannabinol. The second was diagnosed at 22 years of age when he presented with headaches, fever, neuropsychiatric manifestation, and multiple neurological dysfunctions and was alive as of 2020, representing the oldest patient in the literature harboring this diagnosis, followed by our subject in this current report (5).

The linkage between fever and acute neurological decompensation is unique to many neurometabolic disorders. In PEBEL, spontaneous hydration of NADH or NADPH is more pronounced during low-pH or high-temperature states, which impose an overload of neurotoxic metabolites on their already deficient NAD(P)HX repair system, explaining the sensitivity of these patients to febrile illnesses.

The clinical stability and improvement in our patient coincided with the commencement of the mitochondrial cocktail, which probably supported mitochondrial function in the presence of an impaired repair system and secondary mitochondrial dysfunction. Vitamin B3 (Niacin) supplementation restores the NAD+ pool caused by the impaired epimerization of NAD(P)HX, which has been associated with positive therapeutic effects in NAXE-related diseases (5, 14).

## 4 Conclusion

In the case report, a missense homozygous variant in the *NAXE* gene is described in a South African patient. Unlike most other cases presented in the literature, this case represents a milder phenotype with documented clinical improvement followed by clinical stability with relatively milder residual deficits of cerebellar dysfunction. Supplementation with NAD+ in addition to other mitochondrial cocktails may present a potential therapeutic and supportive option to halt or reduce disease progression.

## Data availability statement

The original contributions presented in the study are included in the article/Supplementary material, further inquiries can be directed to the corresponding author.

## Ethics statement

The requirement of ethical approval was waived by spreadsheet. The study meets the requirements of the Human Research Ethics Board (HREB) at Western University, London, Canada for the studies involving humans because spreadsheet. The studies were conducted in accordance with the local legislation and institutional requirements. The participants provided their written informed consent to participate in this study. Written informed consent was obtained from the individual(s) for the publication of any potentially identifiable images or data included in this article.

## Author contributions

MA carried out the data review and drafted the manuscript. CP and AP are the primary treating physicians who also guide through reviewing and editing the manuscript. KT participated in choosing and commenting about the radiological studies. CR helped with molecular interpretation. All authors read and approved the manuscript.

## Footnotes

^1^ https://www.ebi.ac.uk/ProtVar/query?search=Q8NCW5%20K245Q

^2^ https://biosig.lab.uq.edu.au/dynamut/
