# Supplementary figures and images for: Long-term follow-up of an attenuated presentation of NAXE-related disease, a potentially actionable neurometabolic disease: a case report
Source: Front Neurol. 2024 Feb 14;15:1204848. doi: 10.3389/fneur.2024.1204848 (PMC10899487; doi:10.3389/fneur.2024.1204848)

Supplementary Data

**
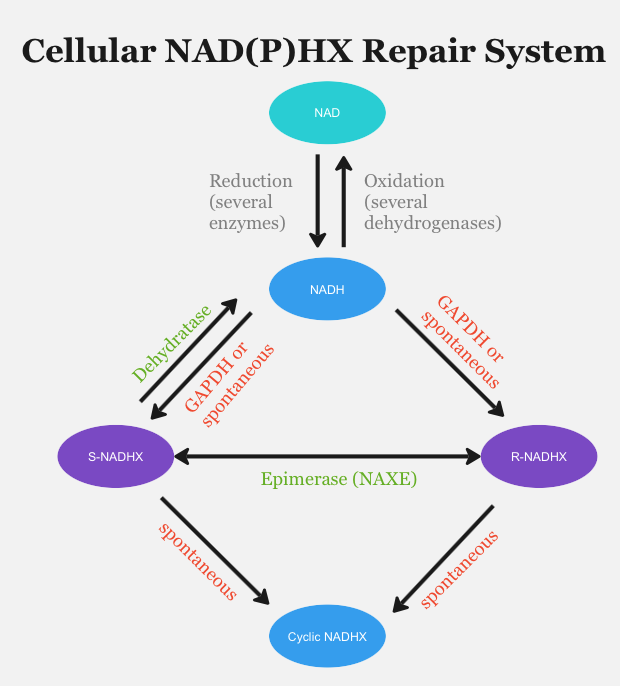
**

**Supplementary Figure 1.** Cellular NAD(P)HX Repair System

Supplement: Supplementary file 1 [file Data_Sheet_1.docx]
