# Supplementary material for: Long-term follow-up of an attenuated presentation of NAXE-related disease, a potentially actionable neurometabolic disease: a case report
Source: Front Neurol. 2024 Feb 14;15:1204848. doi: 10.3389/fneur.2024.1204848 (PMC10899487; doi:10.3389/fneur.2024.1204848)

PEBEL

Mutation effects on Protein structure and function

GENOMIC								PROTEIN						ANNOTATIONS
Chr.	Coordinate	ID	Ref.	Alt.	Gene	Codon (strand)	CADD	Isoform	Protein name	AA pos.	AA change	Consequence(s)	EVE	Click for details
1	156593950		A	C	NAXE	Aaa/Caa (+)	28.9	can Q8NCW5	NAD(P)H-hydrate epim...	245	Lys/Gln	missense		

Table Legends

EVE score colour	CADD phred-like score colour		Annotations
Benign	< 15.0	Likely benign	Functional Information
Pathogenic	15.0 to 19.9	Potentially deleterious	Population Observation
Uncertain	20.0 to 24.9	Quite likely deleterious	Structures
	25.0 to 29.9	Probably deleterious	
	> 29.9	Highly likely deleterious	

https://www.ebi.ac.uk/ProtVar/query?search=Q8NCW5%20K245Q

ID	Substitution	MutPred2 score	Remarks	Affected PROSITE and ELM Motifs
NNRE_HUMAN	K245Q	0.779	-	ELME000239
**Molecular mechanisms with P-values <= 0.05**			**Probability**	**P-value**
Loss of Catalytic site at K245			0.20	0.01
Loss of Methylation at K245			0.13	0.02

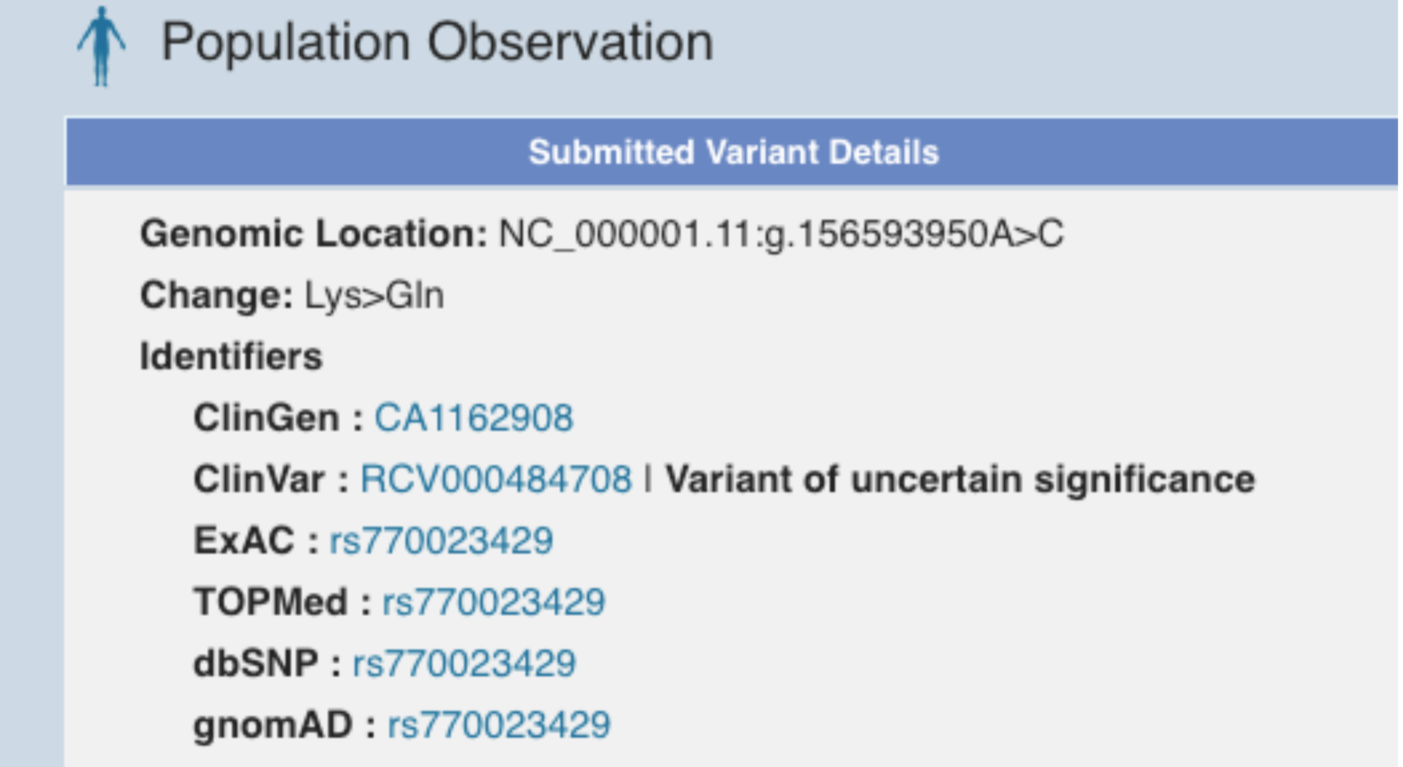
Population Observation
Submitted Variant Details
Genomic Location: NC_000001.11:g.156593950A>C
Change: Lys>Gln
Identifiers
ClinGen : CA1162908
ClinVar : RCV000484708 | Variant of uncertain significance
ExAC : rs770023429
TOPMed : rs770023429
dbSNP : rs770023429
gnomAD : rs770023429

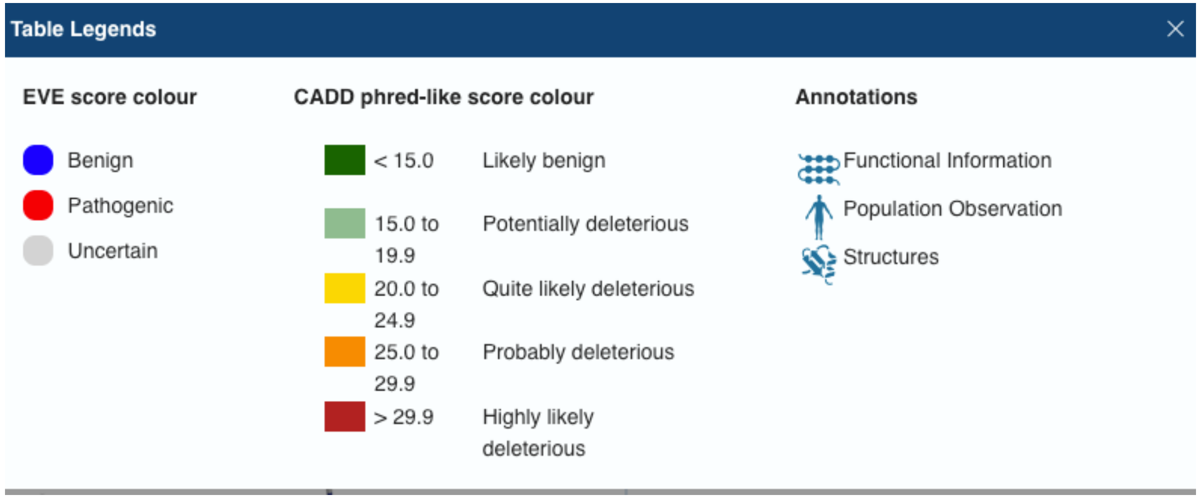
Table Legends
EVE score colour
Benign
Pathogenic
Uncertain
CADD phred-like score colour
< 15.0
Likely benign
15.0 to 19.9
Potentially deleterious
20.0 to 24.9
Quite likely deleterious
25.0 to 29.9
Probably deleterious
> 29.9
Highly likely deleterious
Annotations
Functional Information
Population Observation
Structures

https://www.pantherdb.org/tools/csnpScore.do

Gene List Analysis | Browse | Sequence Search | cSNP Search | Keyword Search

EVOLUTIONARY ANALYSIS OF CODING SNPS

To view information for this subfamily/family, you can search for this family via browser PANTHER HMM Classification file or ftp tool here.

Export results

PANTHER HMM: NAD(P)H-HYDRATE EPIMERASE (PTHR13232)

substitution	preservation time	Message	Pdel
K245Q	1628	probably damaging	0.89

PSEP (position-specific evolutionary preservation) measures the length of time (in millions of years) a position in current protein has been preserved by tracing back to its reconstructed direct ancestors. The longer a position has been preserved, the more likely that it will have a deleterious effect. We convert this to a probability of deleterious effect (Pdel) from results on the HumVar benchmark. We convert this Pdel to a qualitative prediction as follows: "probably damaging" (time > 450my, corresponding to a false positive rate of ~0.2 as tested on HumVar), "possibly damaging" (450my > time > 200my, corresponding to a false positive rate of ~0.4) and "probably benign" (time < 200my).

- Pejaver V, Urresti J, Lugo-Martinez J, Pagel KA, Lin GN, Nam H, Mort M, Cooper DN, Sebat J, Iakoucheva LM, Mooney SD, Radivojac P. Inferring the molecular and phenotypic impact of amino acid variants with MutPred2. *Nat. Commun.* 11, 5918 (2020)

GENOMIC								PROTEIN						ANNOTATIONS
Chr.	Coordinate	ID	Ref.	Alt.	Gene	Codon (strand)	CADD	Isoform	Protein name	AA pos.	AA change	Consequence(s)	EVE	Click for details
1	156593950		A	C	NAXE	Aaa/Caa (+)	28.9	can Q8NCW5	NAD(P)H-hydrate epim...	245	Lys/Gln	missense		

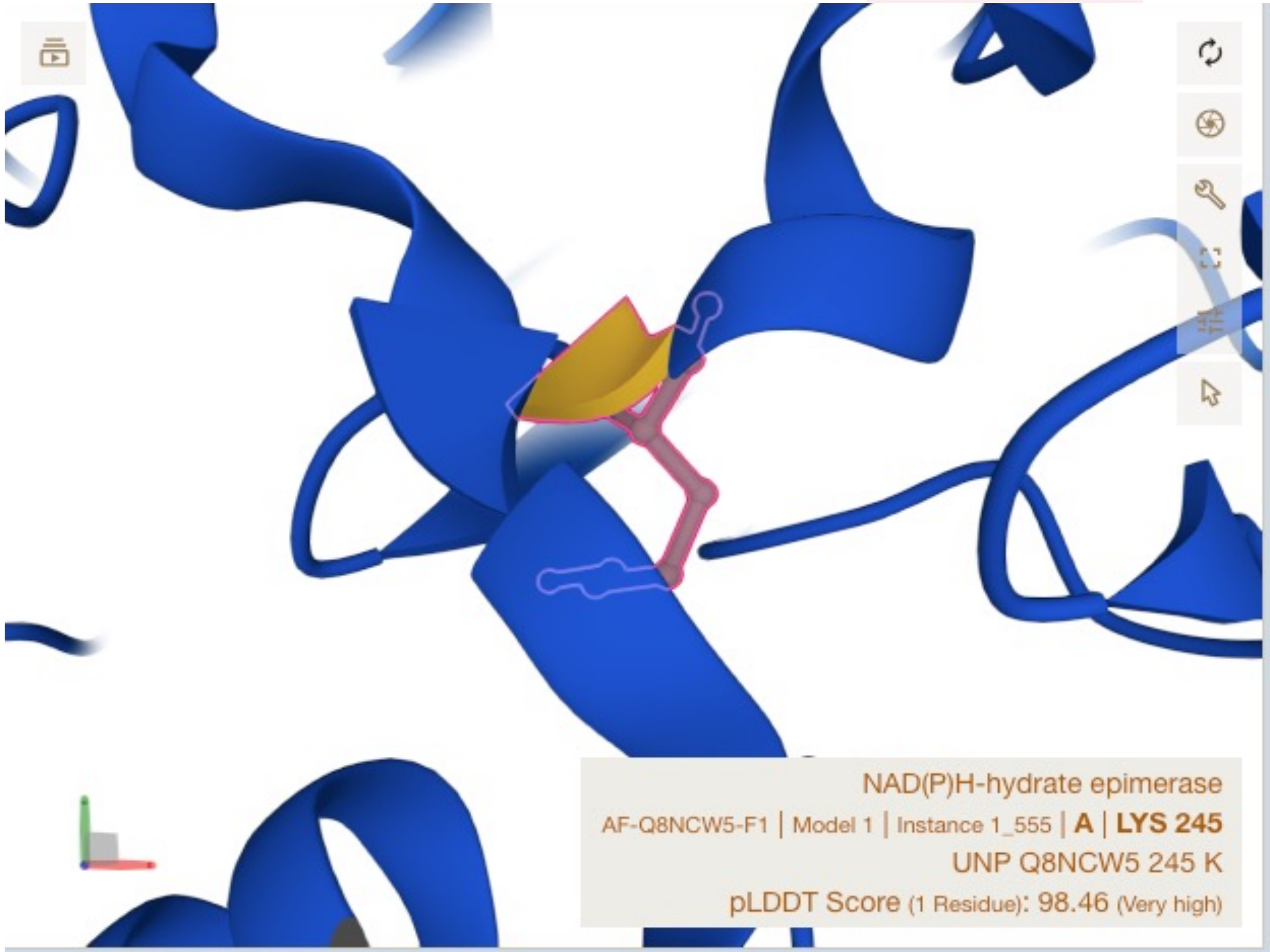

EVE (Evolutionary model of Variant Effects) score. Source: PubMed PMID 34707284

Table Legends

EVE score colour
- Benign
- Pathogenic
- Uncertain

CADD phred-like score colour

< 15.0	Likely benign
15.0 to 19.9	Potentially deleterious
20.0 to 24.9	Quite likely deleterious
25.0 to 29.9	Probably deleterious
> 29.9	Highly likely deleterious

Annotations
- Functional Information
- Population Observation
- Structures

Structures

Image zoomed to show variant location in green
Reference amino acid shown

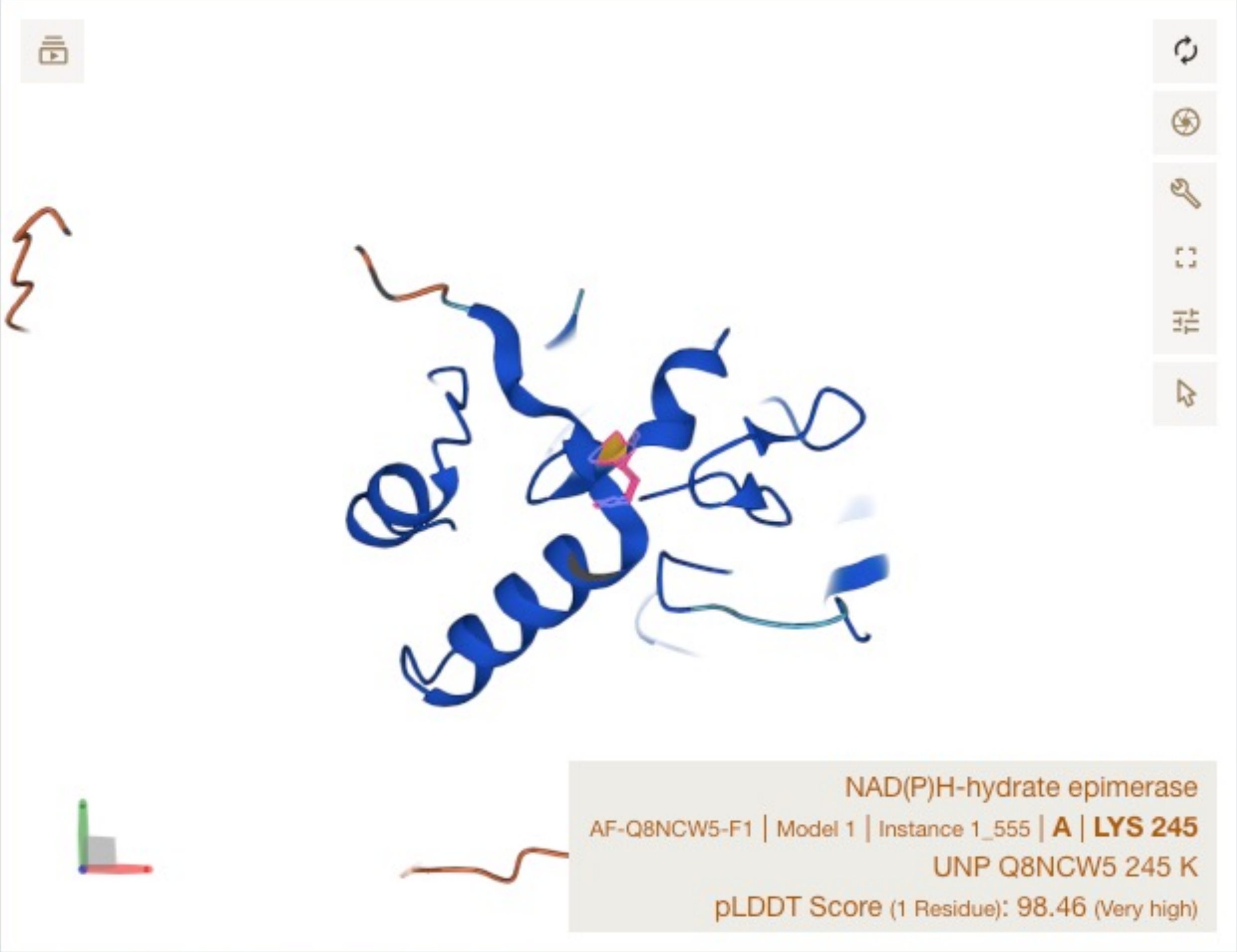

Click variant to see surrounding residues
Click white space to zoom out to whole structure

Predicted Structure			
Source	Identifier	Position	Pockets
AlphaFold	AF-Q8NCW5-F1	245	N/A

Model Confidence

- Very high (pLDDT > 90)
- Confident (90 > pLDDT > 70)
- Low (70 > pLDDT > 50)
- Very low (pLDDT < 50)

Predicted Align Error

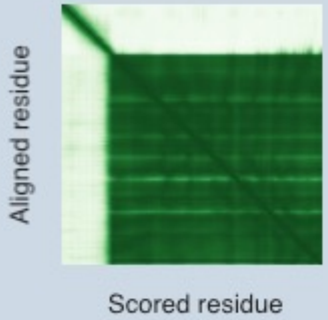

AlphaFold produces a per-residue confidence score (pLDDT) between 0 and 100. Some regions with low pLDDT may be unstructured in isolation.

The colour at position (x, y) indicates AlphaFold's expected position error at residue x, when the predicted and true structures are aligned on residue y.
This is useful for assessing inter-domain accuracy.

DynaMut - Prediction Outcomes

Info! Your results will be available for 7 days after the job is processed. ×

Run another prediction

Submission details

Wild-type: **LYS** *Position:* **245** *Mutant:* **GLN** *Chain:* **A**

ΔΔG Predictions | Interatomic Interactions | Deformation and Fluctuation Analysis

Prediction Outcome

ΔΔG: -0.403 kcal/mol (Destabilizing)

NMA Based Predictions

ΔΔG ENCoM: -0.422 kcal/mol (Destabilizing)

Other Structure-Based Predictions

ΔΔG mCSM: -1.571 kcal/mol (Destabilizing)

ΔΔG SDM: -0.810 kcal/mol (Destabilizing)

ΔΔG DUET: -1.628 kcal/mol (Destabilizing)

Δ Vibrational Entropy Energy Between Wild-Type and Mutant

$\Delta\Delta S_{Vib}$ ENCoM: 0.528 kcal.mol^{-1}.K^{-1} (Increase of molecule flexibility)

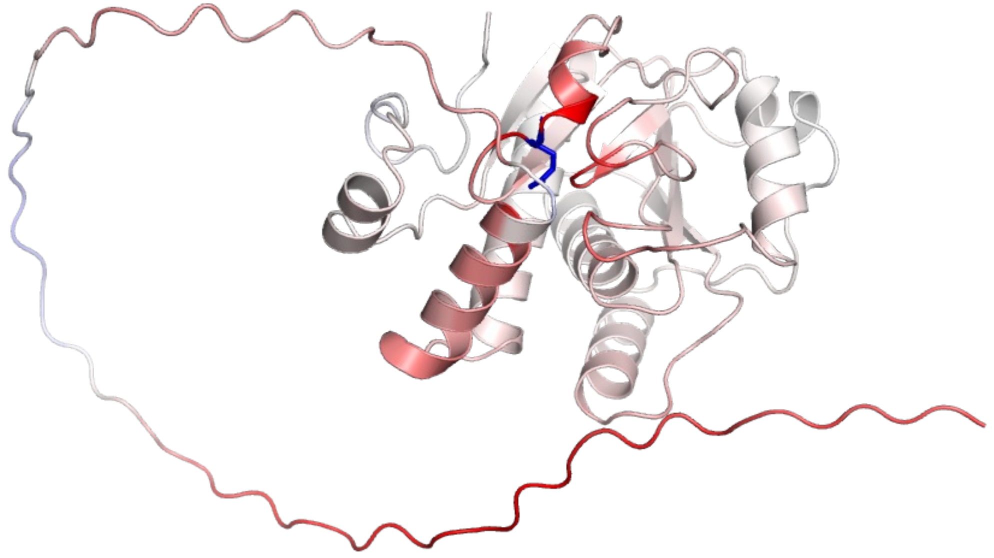

Amino acids colored according to the vibrational entropy change upon mutation. **BLUE** represents a rigidification of the structure and **RED** a gain in flexibility

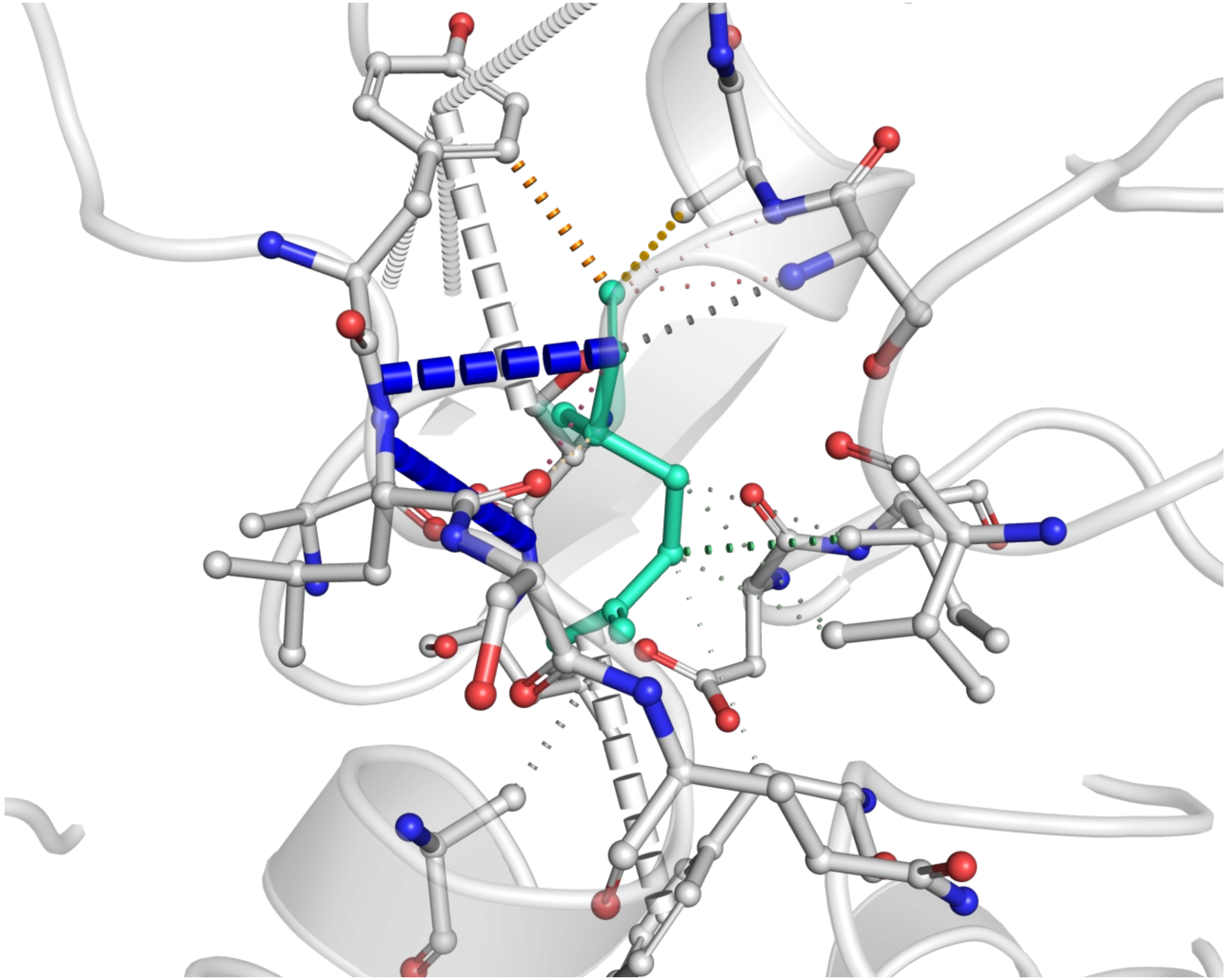

Interactomic Interactions of **MUTANT** residue

Color definition for contacts ▲

Bond Type	Color
Hydrogen bonds	
Water mediated hydrogen bonds	
Weak hydrogen bonds	
Water mediated weak hydrogen bonds	
Halogen bonds	
Ionic interactions	
Metal complex interactions	
Aromatic contacts	
Hydrophobic contacts	
Carbonyl contacts	

Ensemble NMA of Wild-type and Mutant

Wild-type and Mutant sequence were extracted from their respective 3D structures and then aligned. The results of normal mode data for each of the sequences are displayed below.

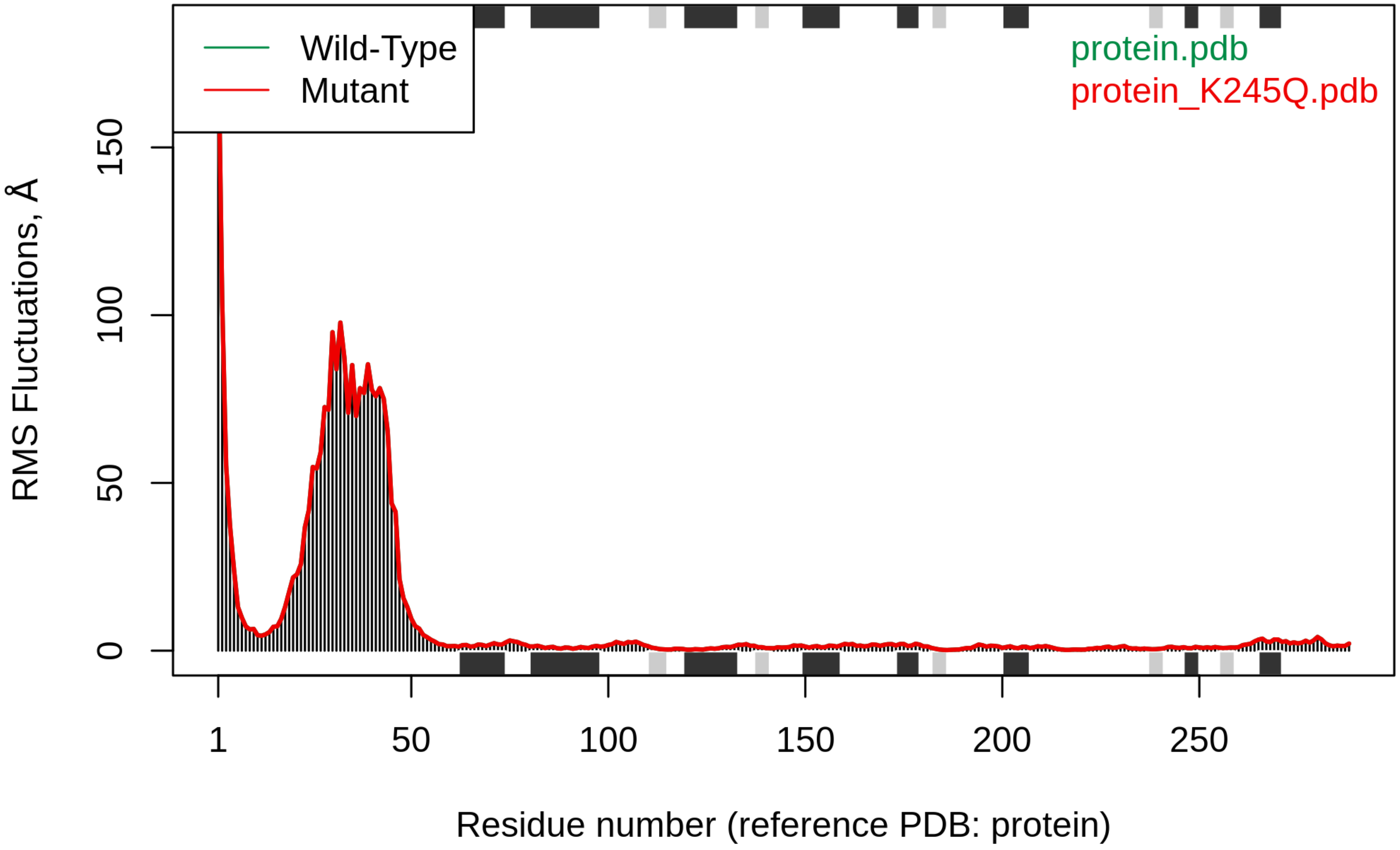

Results of ensemble NMA on **WILD-TYPE** and **MUTANT**

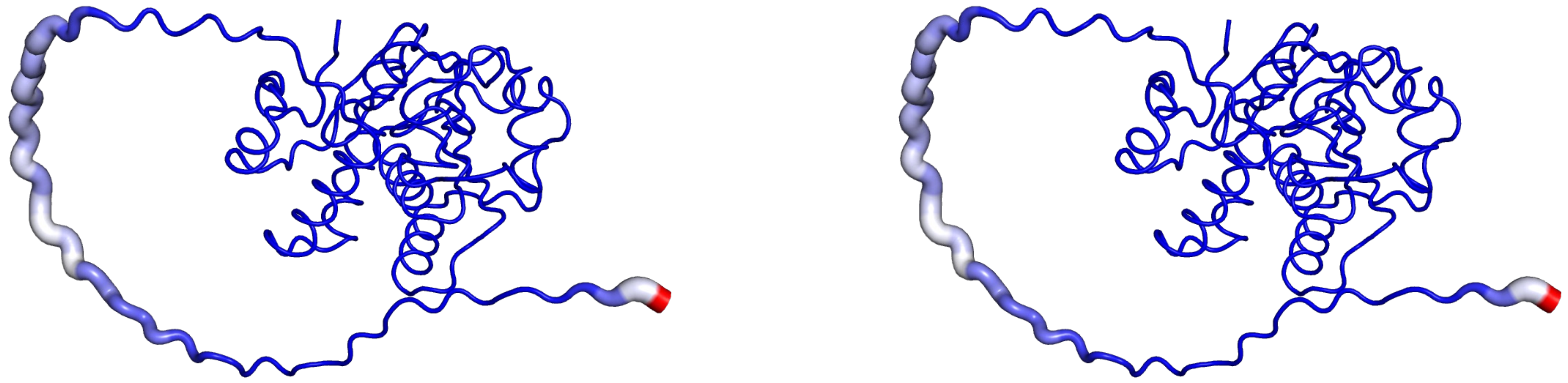

Supplement: Supplementary file 2 [file Data_Sheet_2.PDF]
